# Sex-Related Differences in Immunotherapy Outcomes of Patients with Advanced Non-Small Cell Lung Cancer

**DOI:** 10.3390/curroncol31110544

**Published:** 2024-11-20

**Authors:** Sara Frida Cohen, Diane Cruiziat, Jeremy Naimer, Victor Cohen, Goulnar Kasymjanova, Alan Spatz, Jason Agulnik

**Affiliations:** 1 Anatomy and Cell Biology, Faculty of Science, McGill University, Montreal, QC H3A 0C7, Canada; sara.cohen2@mail.mcgill.ca (S.F.C.); diane.cruiziat@mail.mcgill.ca (D.C.); jeremy.naimer@mail.mcgill.ca (J.N.); victor.cohen@mcgill.ca (V.C.); alan.spatz@mcgill.ca (A.S.); jason.agulnik@mcgill.ca (J.A.); 2 Anna and Peter Brojde Lung Cancer Centre, Jewish General Hospital, Montreal, QC H3T 1E2, Canada; 3 Department of Pathology, Jewish General Hospital, Montreal, QC H3T 1E2, Canada

**Keywords:** mNSCLC, immune checkpoint inhibitors (ICIs), sex-related differences

## Abstract

Background: Immunotherapy with ICIs has revolutionized the treatment for NSCLC. The impact of sex on treatment outcomes remains unclear. The aim of this study was to evaluate sex-related differences in immunotherapy outcomes in a real-world population of NSCLC patients. Methods: Demographics, clinical, pathological characteristics, and treatment-related variables were analyzed to understand the differences in efficacy and safety outcomes in relation to sex. Results: 174 advanced NSCLC patients receiving first-line ICIs, either alone or in conjunction with chemotherapy, were included. No differences based on gender were observed in PFS and OS. Prognostic factors for OS and PFS included liver metastases and CRP levels at treatment discontinuation (TD). IrAE-related TD occurred at a significantly higher rate in females. GI toxicity, including hepatitis and colitis, was predominantly observed in females, whereas pneumonitis was the most frequent irAE leading to TD in males. Conclusions: Despite no significant differences based on gender being observed in survival outcomes, our study showed that female patients with advanced NSCLC receiving ICIs are at a substantially greater risk of severe symptomatic irAEs and TD. This finding indicates that broad-based sex differences could potentially exist and emphasizes the need for further investigations into the role played by gender in immunity and cancer immunotherapy treatment.

## 1. Introduction

Immunotherapy with immune checkpoint inhibitors (ICIs), including immune checkpoint antibodies targeting the PD-1 (programmed death protein)/PD-L1 (programmed death protein ligand) and CTLA-4 (cytotoxic T-cell lymphocyte antigen 4) pathways, has revolutionized cancer treatment and extended survival in patients with advanced non-small cell lung cancer (NSCLC). In recent years, there has been increasing interest in evaluating whether sex differences may have an impact on efficacy and safety outcomes in cancer patients undergoing immunotherapy. A growing body of literature suggests that there may be differences in how male and female patients respond to immunotherapy, which can have implications on treatment outcomes and strategies. Despite this, however, sex as a biological variable has not been meaningfully considered in standard cancer treatment or clinical practice guidelines, highlighting the need for increased awareness and recognition of sex differences in both clinical management and cancer research [1].

It is known that males and females differ in their immunological responses to foreign and self-antigens and show variations in innate and adaptive immune responses [2]. Females typically express more robust reactions because of more vigorous B lymphocyte effects on various antigens and higher production of interferons in macrophages and dendritic cells [2,3]. Additionally, males and females may have different genetic, hormonal, and immune system profiles, which can influence cancer development and response to treatment. Sex hormones play a very important role in regulating the immune system. In some types of lung cancers, estrogens and androgens affect the number and function of immune cells, modify PD-L1 expression in cancer cells, and promote immune escape, although the precise mechanisms of these occurrences remain to be elucidated. Sex chromosomes are yet another key determinant of sex differences in both innate and adaptive immunity. The X chromosome is known to contain a large number of immune-related genes. These genes encode for proteins implicated in immune regulation, including cytokine receptors and important transcription factors that are essential for the development of regulatory T-cells that function to suppress an assortment of physiological and pathological immune responses. X-chromosome inactivation, the epigenetic mechanism that causes the silencing of one X chromosome in females, may be an important contributing factor to these sex differences [4,5,6].

The extensiveness of these recognized differences in immunity has led to important research aiming to determine if there are differences in outcomes between females and males with lung cancer who are treated with ICIs [7,8,9,10]. Several meta-analyses have been conducted with varying results. Meta-analyses carried out by Conforti et al. suggested that male patients derive greater benefits from ICIs than female patients. For example, a meta-analysis of clinical trials indicated that males had a slightly better overall survival benefit from ICIs compared to females [8]. In contrast, other studies presented conflicting results showing no significant difference in immunotherapy outcomes [7,10]. However, these meta-analyses of multiple cancer types are based on subgroup hazard ratios of published clinical trials, which may introduce bias due to a lack of analysis of individual patients or features that differ in their distributions between the sexes. And as such, there is yet no conclusive data on gender differences and cancer immunotherapy in patients with solid malignancies, including advanced NSCLC.

The aim of this retrospective cohort study was to evaluate sex-related differences in immunotherapy outcomes in a real-world population of NSCLC patients treated with immune checkpoint inhibitors, offering insights to enhance patient care. We hypothesized that females were more likely to experience a higher immune-related adverse events (irAEs) burden compared with male patients due to their elevated immune response.

## 2. Materials and Methods

### 2.1. Study Design and Data Sources

This comprehensive retrospective real-world cohort study analyzed data from the Anna and Peter Brojde Lung Cancer Centre database containing structured data derived from electronic health records and unstructured data extracted from physicians’ notes and other documents. Patient charts were accessed for inclusion in this study as part of a CIUSSS West-Central Montreal Research Ethics Board-approved protocol (Protocol Number 2024-3895), in an anonymized fashion, in concordance with the Declaration of Helsinki. Information was collected for each of the treated patients, such as demographics (DOB, age, sex, smoking status, ECOG PS), clinicopathological information (histopathology of lung cancer, date of diagnosis, baseline metastases, PD-L1 status, laboratory results including blood pre-inflammatory factors, i.e., C-reactive protein (CRP), white blood cells (WBCs), and treatment history (type of treatment received, start/end date of treatment, response to treatment, duration of treatment, reason to discontinue treatment).

### 2.2. Study Population

Eligible patients included patients with metastatic NSCLC treated at the Jewish General Hospital, Montreal, QC, Canada, between 2016 and 2022, who met the following criteria: diagnosis of advanced or metastatic NSCLC, ECOG PS 0-1, and who received at least 1 cycle of anti-PD1 or anti-PD-L1 ICIs with or without chemotherapy in the first-line setting.

### 2.3. Outcomes

The primary endpoints of interest were to evaluate sex-related differences in efficacy outcomes in patients receiving immunotherapy (objective response rates (ORRs), median progression-free survival (mPFS), overall survival (OS) outcomes, and mortality rate. Secondary endpoints included the assessment of the prognostic value of C-reactive protein (CRP) and comparison of irAEs between male and female sexes. Information on AE grades was not always available; however, the available data on the incidents related to the AEs, including approaches used for their management, were described.

### 2.4. Data Analysis and Statistical Methods

Statistical analyses and the creation of graphics were performed with Graphpad PRISM version 7 and STATA version 18. All statistical analyses were carried out using the SPSS software, version 24.0 (SPSS, Chicago, IL, USA). *p*-values of 0.05 or less were considered significant. The tumor response was determined according to the Response Evaluation Criteria in Solid Tumors (RECIST 1.1) guidelines and divided into two categories: (1) the objective response defined as complete response (CR) and partial response (PR) and (2) the disease control defined as CR, PR, or stable disease (SD). The statistical methods employed to analyze sex-based differences in outcomes included Chi-square tests for categorical variables and Kaplan–Meier analysis for survival outcomes such as progression-free survival (PFS) and overall survival (OS). Cox proportional hazards models were used to assess the impact of sex on PFS and OS, adjusting for confounding variables. Confounding factors included in the analysis were age, smoking status, ECOG performance status, CRP, WBC, type of therapy (ICIs ± chemotherapy), pre-existing autoimmune diseases, and presence of liver metastasis, all of which were adjusted for in the multivariate analysis. These adjustments allowed us to isolate the effect of sex on treatment outcomes while accounting for potential biases.

Mortality is the number of patients who die at a given time in a study population and was calculated using the live table. Toxicity was assessed according to the Common Terminology Criteria for Adverse Events (version 5.0).

## 3. Results

### 3.1. Study Population

The study population consisted of 174 patients with advanced NSCLC treated with immunotherapy in the first-line setting, either alone or in combination with chemotherapy. The mean age of the study cohort patients was 70 years (SD 9.0). All 174 patients were PS 0-1 and were eligible for treatment with ICIs ± chemotherapy. Of this group, 85 patients were female and 89 were male. Patient characteristics are presented in Table 1.

Females were younger in age, were more likely to be non-smokers, and have non-squamous tumors (81% vs. 67%). Genetic testing was performed in 162 patients: no cases with targetable EGFR mutations were detected, 7 patients were found to have BRAF Non(V600E) mutations, and 44 patients had non-targetable KRAS mutations. Additionally, the prevalence of pre-existing autoimmune diseases, including rheumatoid arthritis, Raynaud syndrome, and psoriasis, was significantly higher in females (18%) compared to males (4.7%). Female patients exhibited a higher baseline CRP level (50.8) compared to males (34.5), *p*-value 0.046.

### 3.2. Efficacy Outcomes Based on Gender

**Figure 1:**
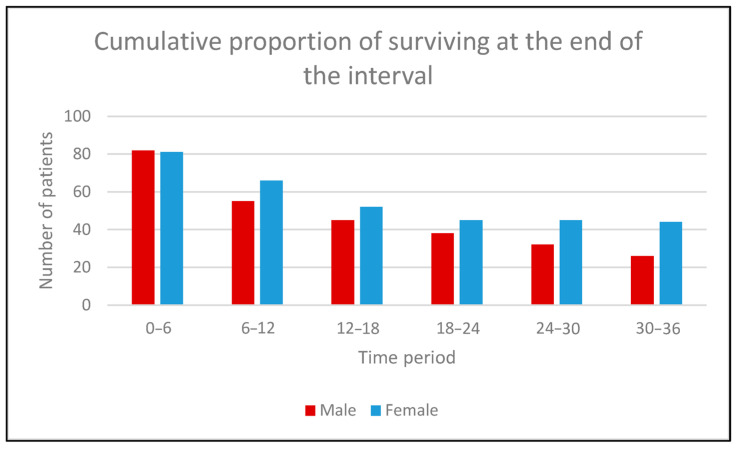
Cumulative proportion of surviving at the end of the interval.

Mean duration of treatment was 8.9 (range: 1–59) months. When assessing response to treatment, the disease was successfully controlled (CR/PR/SD) in 121 (69.5%) patients: 60 (70.6%) males and 61 (68.5%) females. Survival analysis was conducted on 174 patients. Mean follow-up was 22.4 (range 1–87) months. During this follow-up, 115 death events occurred. Mortality rate was not statistically different among males and females. However, females tended to do better compared to their male counterparts, with more female patients having not reached the terminal event for each of the defined time intervals (Figure 1).

**Figure 2:**
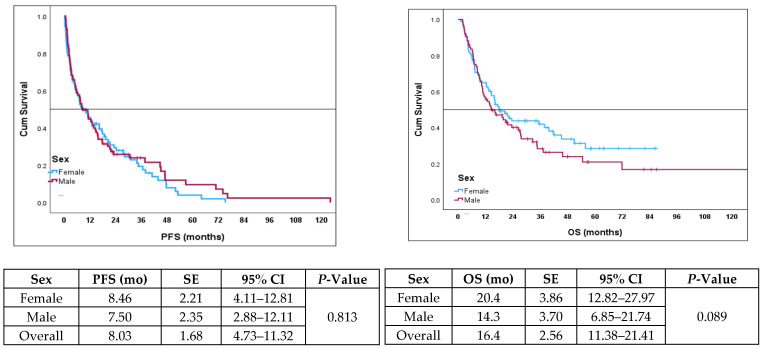
OS and PFS.

In Figure 2, the Kaplan–Meier survival curves illustrate the progression-free survival (PFS) and overall survival (OS) for both females and males. Notably, the median OS was 16.4 months, with females exhibiting 20.4 and males 14.3 months. The median PFS was 8.4 months for females and 7.5 months for males, yet the observed difference did not reach statistical significance.

The survival rates of males and females who discontinued treatment either due to irAEs or due to progression are similar. This suggests that sex does not appear to be a significant factor influencing survival (Table 2).

### 3.3. Prognostic Factors for PFS and OS

Univariate analysis of survival revealed that CRP, age, and presence of liver metastases at the time of diagnosis emerged as significant predictors. Other variables examined demonstrated no impact on survival outcome (Table 3).

We then further explored the effect of variables of interest on PFS and OS among males and females. All the variables presented in Table 2 were included in the backward stepwise regression analysis (Table 4). To adjust for the baseline differences between males and females, we used stratification by sex. The analysis identified CRP level at the time of discontinuation and the presence of liver metastasis as significant predictor factors of PFS. This finding suggests that abnormal CRP and the presence of liver metastases are significantly associated with increased risk of progression in both sexes. For OS in both sexes, we identified the CRP variable and PD-L1 as significant predictive factors: elevated CRP increases the risk of dying, while increased PD-L1 scores significantly improve survival. Age and pre-existing liver diseases may have some effect on survival; however, it is not strong enough as the association is not statistically significant. Of note, at the time of discontinuation due to disease progression, males exhibited significantly higher levels of CRP compared to females (Table 4 and Table 5).

### 3.4. Treatment Details and AE Data

Out of 174 patients, 17 (10%) remained on treatment at the dataset lock, while 20 (11%) had completed the full 2-year treatment. In 137 (79%), TD occurred due to either disease progression or treatment-associated adverse events. The proportion of patients continuing treatment and those who completed the full 2-year treatment was similar among males and females. However, females had a significantly increased risk of severe toxicity and 28% less risk of progression compared to males (Table 6). GI toxicity, including hepatitis and colitis, was predominantly observed in females, whereas ir-pneumonitis was more prevalent in males (Table 7).

## 4. Discussion

This study described the real-world sex-related differences in the efficacy and safety of immunotherapy in patients with advanced NSCLC. Our findings showed that females at baseline tended to be non-smokers, have non-squamous histology, and more often suffered from autoimmune diseases compared to the male sex. These particular observations are reassuring in that they are consistent with the typical demographics of an advanced lung cancer patient population, with lung cancer seen more commonly in males, largely due to higher smoking rates, and an increased prevalence of autoimmune diseases in females has been well recognized in the medical literature for more than 100 years [11,12,13].

Efficacy outcome analysis in our cohort demonstrated no differences in mPFS, OS, and mortality rate among males and females receiving ICI therapy. There are several potential explanations for this. Biological factors, such as hormonal influences and immune system differences, have been postulated to affect cancer outcomes based on sex, particularly in immunotherapy settings. However, these factors might not always translate into measurable differences in survival in smaller cohorts or under specific treatment regimens. Additionally, confounding variables such as comorbidities, treatment modalities, and tumor biology may obscure sex-related effects in real-world populations. Other studies have suggested that male patients may respond better to ICIs than female patients. In contrast to our findings, a meta-analysis of Phase II and III checkpoint inhibitor trials suggests that checkpoint inhibitors enhance overall survival and progression-free survival for both male and female patients across cancer types such as melanoma, urothelial, and non-small cell lung cancer. However, the improvements are notably more pronounced in males than females [12,14,15,16].

While some report better outcomes in males, others find no significant difference or even better outcomes in females [3,6]. Gandhi et al., in the KEYNOTE 189 trial, evaluated pembrolizumab combined with standard-of-care (SOC) chemotherapy (n = 410) versus SOC chemotherapy alone (n = 206) as a first-line treatment for patients with NSCLC. The study included 363 males and 253 females. Results showed a significant overall survival benefit from immunotherapy in females (HR 0.29; 95% CI 0.19–0.44) compared to males (HR 0.70; 95% CI 0.50–0.99) [17]. In KEYNOTE 407, when first-line pembrolizumab was compared to SOC chemotherapy, females had a significant advantage compared to males despite the fact that females made up only 18.6% of the study cohort [18]. Wallis et al. performed a meta-analysis of 23 trials. The authors concluded that both male and female patients showed an OS benefit from ICI treatment, with no significant difference in the OS advantage between genders (*p* = 0.60).

The underlying reasons for these discrepancies are not fully understood but may involve variations in tumor immunogenicity, hormone levels, and immune system functioning between sexes. The differences in outcomes in these studies might also be attributed to the under-representation of females [7]. In our study, both sexes were equally represented, and we found no statistically significant difference in treatment outcomes among females and males.

Our results demonstrated that CRP values both at the time of diagnosis (pretreatment) and at the time of TD were important predictors of disease progression and poor survival outcomes. McAllister and colleagues have shown that inflammation within the tumor microenvironment accelerates cancer progression by driving cell proliferation, angiogenesis, and cancer cell migration [19]. We recognize that CRP is a nonspecific marker of inflammation and can be influenced by factors beyond cancer, such as chronic obstructive pulmonary disease (COPD), bronchiectasis, autoimmune diseases, and smoking—all of which are prevalent in our patient population and particularly in men. However, it has been shown that elevated baseline CRP is associated with poor response to chemotherapy and adverse disease outcomes in cancer patients, including advanced NSCLC. Suzuki et al. found a significant link between high pretreatment CRP levels and reduced OS in patients with metastatic renal cell carcinoma treated with nivolumab. They further noted that a CRP reduction of ≥25% during ICI therapy was associated with a better treatment response [20]. In NSCLC patients treated with the PD-1 inhibitor nivolumab, Oya et al. identified elevated CRP as an independent predictor of decreased PFS [21]. Moreover, it has been suggested by Miceli, R and colleagues that CRP plays a key role in ICI-mediated antitumor immune response [22].

An abnormal CRP in our study was significantly associated with increased risk of progression in both sexes. For OS in both males and females, we identified the CRP variable as a significant negative predictive factor; elevated CRP was associated with an increased risk of dying. Of note, at the time of discontinuation due to disease progression, males exhibited significantly higher levels of CRP compared to females, suggesting a possible significant sex-related predictive impact on survival outcomes in lung cancer patients treated with ICIs. Further validation using larger sample sizes (and across multiple cancer types) will be necessary to determine if sex-related differences in serum CRP levels (and perhaps even other cytokines) will be useful predictive biomarkers for immunotherapy. If it is established that serum CRP and other cytokines have a significant sex-related predictive impact on efficacy and survival outcomes, these results could potentially pave the way for new ICI combinations designed according to baseline levels and early changes in these cytokines and stratified by sex.

Finally, in addition to considering the impact of ICI on immune responses and survival outcomes, we have found that irAEs were significantly more frequent in females and often were the main reason for TD, while males more frequently discontinued treatment due to progression of disease. The higher incidence of irAEs in female patients is a notable finding with significant clinical implications. Females tend to have a more robust immune response, which may explain their heightened risk of irAEs. Clinicians should be more vigilant when treating female patients with ICIs, implementing proactive strategies for early detection and management of irAEs. This could include more frequent monitoring for symptoms, early intervention, and considering pre-emptive management strategies, such as corticosteroids or other immunosuppressive treatments, in cases where the risk of severe irAEs is high. Tailoring treatment plans to minimize toxicity without compromising efficacy could improve the quality of life and overall treatment outcomes for female patients. Further research is needed to determine if dosage adjustments or modified treatment regimens might help mitigate these risks in women without reducing the efficacy of ICIs.

The types of irAEs were also different among females and males: GI toxicities such as hepatitis and colitis were predominantly observed in females, while pneumonitis was more common among males. Similarly, Miceli, R. and colleagues reported a significant increased risk of irAEs in females [23]. While serious investigation has been given to the difference in irAEs that occur based on the type and dose of checkpoint inhibitors, to date, no studies have considered whether these adverse events may occur differently in males than females.

### Strengths and Limitations

The main strength of the present study is that it was conducted in a real-world setting. Consequently, it provided important insights into the immunotherapy treatment use and outcomes of a NSCLC patient population far more representative of the unselected population than those of clinical trials producing results with enhanced external validity and generalizability. Our study, however, had several important limitations that should be acknowledged. It, of course, carried all the recognized inherent biases of a retrospective single-center study, including incomplete or missing documentation and poorly recorded or absent information. When assessing the secondary endpoint of safety and comparing irAEs between male and female sexes, information on AE grades was not always available in the data. However, the available data on the incidents related to the AEs, including approaches used for their management, were described, serving as a proxy to infer the severity of AEs in our patient population. Future studies should focus on sex-specific immune modulation, looking into how hormonal variations, such as estrogen and testosterone, impact the efficacy and toxicity of immune checkpoint inhibitors (ICIs). Furthermore, larger cohort studies or meta-analyses could help validate findings and determine if sex-based differences should influence dosing strategies or treatment protocols.

## 5. Conclusions

The results from our present real-world experience of patients with advanced NSCLC showed no differences in PFS, OS, and mortality rates among males and females receiving ICI therapy. Our study showed importantly that female patients with advanced NSCLC receiving ICIs are at a substantially increased risk of severe symptomatic irAEs leading to TD. This finding indicates that broad-based sex differences in this context could potentially exist and emphasizes the need for further investigations into the role played by gender in immunity and cancer immunotherapy treatment.

## Figures and Tables

**Table 1:** Patient characteristics. 1—PD-L1 was undetermined in 3 patients; 2—immune checkpoint inhibitors; 3—chemotherapy; 2 patients were missing baseline CRP; 4—N = 162.

Characteristics			Male N = 85	Female N = 89	*p*-Value
Age (mean; range)			71 (42–87)	68 (41–89)	0.097
Smoking history (n/%)	Former/current smoker	n = 153	79 (92.9)	74 (83.0)	0.039
	Non-smoker	n = 21	6 (7.1)	15 (17.0)	
PD-L1: (n/%) ^1^	≥50	n = 101	50 (61.0)	51 (58.6)	0.755
	<50	n = 42	32 (39.0)	36 (41.4)	
Histology	Adenocarcinoma	n = 112	48 (56.0)	64 (71.9)	0.028
	Large cell carcinoma	n = 17	9 (11.1)	8 (9.0)	
	Squamous	n = 45	28 (32.9)	17 (19.1)	
Genetic testing ^4^	EGFR	n = 0/162	0	0	0.489
	Non(V600E)	n = 7/162	3 (3.6)	4 (4.8)	
	KRAS (nonG12C)	n = 44/162	21 (25.7)	23 (27.0)	
	Negative	n = 111/162	58 (70.7)	53 (62.4)	
Treatment type	Mono-ICI ^2^	n = 80	40 (52.9)	40 (44.9)	0.449
	ICI+CTX ^3^	n = 94	45 (47.1)	49 (55.1)	
Liver metastases at the baseline	Present	n = 10	5 (5.6)	5 (5.6)	0.597
	Absent	n = 164	80 (94.1)	84 (94.4)	
History of autoimmune disease	Present	n = 14	4 (4.7)	16 (18.0)	0.005
	Absent	n = 160	81 (95.3)	73 (82.0)	
CRP mean (SD)		n = 174	34.5 (40.9)	50.8 (61.9)	0.046

**Table 2:** Treatment outcomes by sex.

Treatment Outcomes	Males	Females	*p*-Value
Ongoing (n = 17)	All censored	All censored	n/a ^1^
Completed 2 years (n = 20)	Not reached	Not reached	n/a
Discontinued due to PD (n = 86)	9.40 (7.3–11.4)	13.1 (7.4–18.7)	0.140
Discontinued due to irAEs (n = 51)	16.4 (2.3–30.4)	17.86 (9.7–25.9)	0.984

^1^—not applicable.

**Table 3:** Univariate analysis of OS. 1—C-reactive protein; 2—not reached; 3—immunotherapy with chemotherapy; 4—immunotherapy.

Variables of Interest		OS (mo)	SE	95% CI	*p*-Value
Sex	Female Male	20.40 14.30	3.86 3.79	12.8–27.9 6.8–74	0.089
Smoking	Never Ever	16.13 16.4	8.21 2.85	0.03–32.2 10.7–22.0	0.971
CRP at the start of IO	≤10 mg/L >10 mg/L	35.26 14.50	7.59 1.55	20.3–50.1 11.4–17.5	0.013
CRP ^1^ at the end of treatment	≤10 mg/L >10 mg/L	NR ^2^ 14.53	1.43	11.7–17.3	<0.001
Age	≤65 >65	18.03 16.40	5.71 2.67	6.8–29.23 11.1–21.6	0.057
Treatment	IO+CTX ^3^ IO ^4^	15.83 20.80	2.56 5.80	10.7–20.8 9.41–32.2	0.137
PD-L1	≥50 <50	18.03 20.80	3.87 6.22	10.4–25.6 0.0–24.36	0.464
Histology ^3^	Non-squamous Squamous	18.03 13.86	2.52 3.08	13.1–22.9 7.82–19.9	0.418
Liver metastases	Present Absent	9.23 18.03	4.53 2.77	0.35–18.1 12.6–23.4	0.034
Pre-existing liver disease	Present Absent	15.77 20.40	3.46 8.67	8.9–22.5 3.4–37.48	0.210

**Table 4:** Final Cox regression analysis for OS and PFS.

Variables (Reference)	Exp(B)	95% CI	*p*-Value	Variable (Reference)	Exp(B)	95% CI	*p*-Value
	COX Model for PFS				COX Model for OS		
CRP at the TD (<10 g/L)	1.638	1.10–2.43	0.014	CRP at the TD (<10g/L)	2.68	1.58–4.78	<0.001
Liver metastases (absent)	2.48	1.25–4.94	0.009	Age (<65)	1.43	0.92–2.19	0.106
				PD-L1	0.704	0.62–1.00	0.073
				Pre-existing liver disease	0.655	0.38–1.09	0.113

**Table 5:** CRP level at the end of the treatment. 1—progressive disease; 2—immune-related adverse events.

Treatment Outcomes	Mean CRP (SD)		*p*-Value
	Male	Female	
Completed 2 years (n = 20)	18.1 (35.7)	14.3 (14.0)	0.746
Discontinued d/t PD ^1^ (n = 86)	99.3 (93.5)	53.2 (52.9)	0.009
Discontinued d/t irAEs ^2^ (n = 51)	73.2 (96.8)	57.9 (73.2)	0.540

**Table 6:** Treatment discontinuation.

Reason To Stop	Males	Females	RR ^1^	96% CI	*p*-Value
Ongoing (n = 17)	9 (10.6)	8 (9.0)	1.03	0.50–2.13	0.926
Completed 2 years (n = 20)	9 (10.6)	11 (12.4)	0.95	0.41–2.17	0.913
Discontinued d/t PD (n = 86)	49 (57.6)	37 (41.5)	0.86	061–1.20	0.036
Discontinued d/t irAEs (n = 51)	18(21.2)	33 (37.1)	1.75	1.00–2.82	0.028
Total	85 (100)	89 (100)			

^1^ Relative risk.

**Table 7:** Incidence of irAEs in males and females.

Incidence of irAEs in Males and Females	Male N = 85	Female N = 89
ir-pneumonitis	8 (9.5)	3 (3.4)
ir-colitis	3 (3.6)	11 (12.4)
ir-hepatitis	2 (2.3)	7 (7.9)
ir-thyroiditis	2 (2.3)	8 (8.9)
ir-miocarditis	2 (2.3)	1 (1.1)
other	1 (1.2)	3 (3.4)
No irAEs	67 (78.9)	56 (62.9)

## Data Availability

The data presented in this study are available on request from the corresponding author.

## References

[1] Ma J., Yao Y., Tian Y., Chen K., Liu B. (2022). Advances in sex disparities for cancer immunotherapy: Unveiling the dilemma of Yin and Yang. Biol. Sex. Differ..

[2] Jaillon S., Berthenet K., Garlanda C. (2019). Sexual Dimorphism in Innate Immunity. Clin. Rev. Allergy Immunol..

[3] Klein S.L., Flanagan K.L. (2016). Sex differences in immune responses. Nat. Rev. Immunol..

[4] Haupt S., Caramia F., Klein S.L., Rubin J.B., Haupt Y. (2021). Sex disparities matter in cancer development and therapy. Nat. Rev. Cancer.

[5] Meester I., Manilla-Munoz E., Leon-Cachon R.B.R., Paniagua-Frausto G.A., Carrion-Alvarez D., Ruiz-Rodriguez C.O., Rodriguez-Rangel X., Garcia-Martinez J.M. (2020). SeXY chromosomes and the immune system: Reflections after a comparative study. Biol. Sex. Differ..

[6] Ono M. (2020). Control of regulatory T-cell differentiation and function by T-cell receptor signalling and Foxp3 transcription factor complexes. Immunology.

[7] Wallis C.J.D., Butaney M., Satkunasivam R., Freedland S.J., Patel S.P., Hamid O., Pal S.K., Klaassen Z. (2019). Association of Patient Sex With Efficacy of Immune Checkpoint Inhibitors and Overall Survival in Advanced Cancers: A Systematic Review and Meta-analysis. JAMA Oncol..

[8] Conforti F., Pala L., Bagnardi V., De Pas T., Martinetti M., Viale G., Gelber R.D., Goldhirsch A. (2018). Cancer immunotherapy efficacy and patients’ sex: A systematic review and meta-analysis. Lancet Oncol..

[9] Conforti F., Pala L., Bagnardi V., Viale G., De Pas T., Pagan E., Pennacchioli E., Cocorocchio E., Ferrucci P.F., De Marinis F. (2019). Sex-Based Heterogeneity in Response to Lung Cancer Immunotherapy: A Systematic Review and Meta-Analysis. J. Natl. Cancer Inst..

[10] Botticelli A., Onesti C.E., Zizzari I., Cerbelli B., Sciattella P., Occhipinti M., Roberto M., Di Pietro F., Bonifacino A., Ghidini M. (2017). The sexist behaviour of immune checkpoint inhibitors in cancer therapy?. Oncotarget.

[11] Takahashi T., Iwasaki A. (2021). Sex differences in immune responses. Science.

[12] Chen C., Zhang C., Jin Z., Wu B., Xu T. (2022). Sex differences in immune-related adverse events with immune checkpoint inhibitors: Data mining of the FDA adverse event reporting system. Int. J. Clin. Pharm..

[13] Whitacre C.C., Reingold S.C., O’Looney P.A. (1999). A gender gap in autoimmunity. Science.

[14] Conforti F., Pala L., Goldhirsch A. (2018). Different effectiveness of anticancer immunotherapy in men and women relies on sex-dimorphism of the immune system. Oncotarget.

[15] Ortona E., Pierdominici M., Rider V. (2019). Editorial: Sex Hormones and Gender Differences in Immune Responses. Front. Immunol..

[16] Vavala T., Catino A., Pizzutilo P., Longo V., Galetta D. (2021). Gender Differences and Immunotherapy Outcome in Advanced Lung Cancer. Int. J. Mol. Sci..

[17] Gandhi L., Rodriguez-Abreu D., Gadgeel S., Esteban E., Felip E., De Angelis F., Domine M., Clingan P., Hochmair M.J., Powell S.F. (2018). Pembrolizumab plus Chemotherapy in Metastatic Non-Small-Cell Lung Cancer. N. Engl. J. Med..

[18] Paz-Ares L., Luft A., Vicente D., Tafreshi A., Gumus M., Mazieres J., Hermes B., Cay Senler F., Csoszi T., Fulop A. (2018). Pembrolizumab plus Chemotherapy for Squamous Non-Small-Cell Lung Cancer. N. Engl. J. Med..

[19] McAllister S.S., Weinberg R.A. (2014). The tumour-induced systemic environment as a critical regulator of cancer progression and metastasis. Nat. Cell Biol..

[20] Suzuki K., Terakawa T., Furukawa J., Harada K., Hinata N., Nakano Y., Fujisawa M. (2020). C-reactive protein and the neutrophil-to-lymphocyte ratio are prognostic biomarkers in metastatic renal cell carcinoma patients treated with nivolumab. Int. J. Clin. Oncol..

[21] Oya Y., Yoshida T., Kuroda H., Mikubo M., Kondo C., Shimizu J., Horio Y., Sakao Y., Hida T., Yatabe Y. (2017). Predictive clinical parameters for the response of nivolumab in pretreated advanced non-small-cell lung cancer. Oncotarget.

[22] Riedl J.M., Barth D.A., Brueckl W.M., Zeitler G., Foris V., Mollnar S., Stotz M., Rossmann C.H., Terbuch A., Balic M. (2020). C-Reactive Protein (CRP) Levels in Immune Checkpoint Inhibitor Response and Progression in Advanced Non-Small Cell Lung Cancer: A Bi-Center Study. Cancers.

[23] Miceli R., Eriksson H., Lo Russo G., Alfieri S., Moksnes Bjaanaes M., Pietrantonio F., De Cecco L., Prelaj A., Proto C., Franzen J. (2024). Gender Difference in sidE eFfects of ImmuNotherapy: A possible clue to optimize cancEr tReatment (G-DEFINER): Study protocol of an observational prospective multicenter study. Acta Oncol..

